# The Effect of Organizational Trust on Turnover Intention of Rural Kindergarten Teachers: The Mediating Role of Teaching Efficacy and Job Satisfaction

**DOI:** 10.3390/ijerph191912403

**Published:** 2022-09-29

**Authors:** Yan Zhao, Zhenjie Lu, Xiulan Cheng, Jiaqi Li

**Affiliations:** 1 Faculty of Education, Shaanxi Normal University, Xi’an 710062, China; 2 School of Philosophy and Social Development, Shandong University, Ji’nan 250100, China

**Keywords:** mental health, rural kindergarten teachers, organizational trust, teaching efficacy, job satisfaction, turnover intention

## Abstract

Recent studies have focused on turnover among rural kindergarten teachers. However, none of these studies have shown a clear connection between turnover intention and organizational trust, although there are studies in other areas showing that organizational trust can affect turnover intention. Drawing on a sample of 330 kindergarten teachers in rural areas, this study explores the mechanism of influence between organizational trust and turnover intention with teaching efficacy and job satisfaction as mediators. We found that organizational trust negatively impacted teachers’ turnover intention, and this relationship was mediated by a significant chain mediating effect of teaching efficacy and job satisfaction. The findings enrich knowledge about turnover among rural kindergarten teachers and inspire us to create a more supportive organizational environment against the backdrop of the COVID-19 pandemic to improve job satisfaction and alleviate turnover among rural kindergarten teachers.

## 1. Introduction

Turnover can be an issue in any organization, but it has particular importance in an educational context, since teacher training takes time and resources and since experience and continuity by teachers contribute to a successful school environment. Teachers’ turnover intention is defined as their willingness to leave the organization or quit a job [1], which is a precursor of actually resigning [2,3].

The impact of teacher turnover on education can be devastating [4]. Most studies show that high turnover rates among kindergarten teachers imperil the quality and stability of teaching staff and restrict the all-round development of preschool education in the country [5], which is a serious problem worldwide [6]. High turnover among preschool teachers can damage the establishment of a stable teacher–child relationship. Moreover, it increases emotional pressure on children and reduces the development level of their language expression [7]. Conversely, preschool teacher retention positively impacts children’s behavioral and cognitive development [4].

Notably, there is a lot of mobility among Chinese rural kindergarten teachers [8]. In China, due to the existence of a so-called ‘‘urban–rural two system’’ meaning that the government’s strategic planning prioritizes urban areas for economic development while rural development is to support the urban economy, results in the poorest and least developed areas of China being concentrated in rural central and western China. Thus, it is challenging to recruit highly qualified and talented teachers to work in villages due to the weak financial solvency [9]. Specifically, rural kindergarten teachers often face lower salaries [10] and heavier workloads with long working hours and other job requirements, such as nursing, teaching and home-based education [9]. Additionally, the treatment of early childhood educators in rural China has lagged far behind that of urban schools due to lack of necessary attention and support [9].Therefore, reduction in the resignation rate among rural kindergarten teachers is worthy of attention.

Current studies on turnover focus mainly on the analysis of demographic characteristics, including age [11], gender [12], etc., and also explore the influence of students [13], school size [14], school location [15], teaching subjects [16], school discipline [17] and other working conditions on teachers’ turnover intention. By reviewing previous studies, Simon and Johnson found that poor working conditions, especially social conditions (including interpersonal relationships and school culture), were the main factors that led to teacher turnover [18]. It is essential to note, however, that there are few specialized studies on organizational environmental factors and internal mechanisms that effect the turnover intention in rural kindergarten teachers. Based on this, the present study explores influential factors in turnover intention in rural kindergartens.

Trust is a state of mind where one is willing to accept consequences based on positive expectations of the intentions and actions of others [19]. Trust can exist between different cohorts (e.g., colleagues, managers, organizations) and be analyzed at different levels (e.g., individual-level trust or group-level trust) [20]. Organizational trust is a kind of group-level trust that refers to the rational judgment of individuals to fulfill their responsibilities and trust the organization [21]. Organizational trust in this study means that employees within an organization identify and demonstrate their loyalty to the organization [22]. Studies have shown that employees’ trust in an organization is crucial to the success of the organization, and managers’ trust in employees also has an impact [23]. Employees’ organizational trust can improve operating efficiency and promote organizational development and personal growth [24], as well as predict turnover [25]. There is a strong correlation between job satisfaction and turnover intention. When organizational trust is high, employees’ job satisfaction can be improved [26], and turnover intention can be reduced [27]. In recent years, based on prior research, some researchers are paying attention to teachers’ organizational trust and have found that teachers’ organizational trust is significantly positively correlated with work engagement [28,29]. However, there is still a lack of empirical studies on whether the organizational trust in kindergarten will affect the preschool teachers’ turnover intention.

The concept of teaching efficacy comes from Bandura’s social cognition theory [30]. Existing studies define teaching efficacy as the teachers’ belief that they have the ability to organize and successfully complete specific teaching tasks in a specific teaching situation [31]. Teachers’ teaching efficacy affects their teaching behavior [32] and their views on teaching abilities and creates a self-fulfilling prophecy [33]. Existing research on enterprise employees shows that employees with a high sense of job self-efficacy are fully engaged in their work and less likely to quit [34]. In addition, there is a certain correlation between organizational trust and employee self-efficacy. Individuals with high organizational trust tend to have a more positive work attitude, better work performance, and stronger willingness to cooperate [35]. After that, the employees’ sense of self-efficacy and work output will be improved [36,37]. Research has found that the teaching efficacy in kindergarten teachers can significantly affect teaching quality and educational effect [38]. The teaching efficacy in kindergarten teachers is significantly correlated with organizational consciousness and organizational environment [39]. However, few studies directly prove the relationship between organizational trust and the teaching efficacy in kindergarten teachers. The studies mentioned above can be used as a basis for a hypothesis about the influence of organizational trust on the teaching efficacy in rural kindergarten teachers.

Job satisfaction refers to the employees’ positive or negative attitudes towards their jobs [40] and also the extent to which the experience obtained from work can meet their needs. Studies have shown that if employees think their needs have not been met, they will be dissatisfied with the current work environment and will be more likely to resign [41]. Mature researchers have found a negative correlation between teacher turnover and job satisfaction [42,43]. In addition, existing studies show that in the preschool education context, job satisfaction can predict the actual resignation rate in kindergarten teachers, and improving job satisfaction can reduce turnover [44]. Studies have also shown that employee job satisfaction and organizational trust are significantly correlated [45]. Employees with higher organizational trust achieve higher work performance, are more willing to accept organizational values and show better civic behavior. They are also inclined to accept managers’ views and regard them as learning resources [46] to stimulate creativity and job satisfaction. Although there is no direct research showing the relationship between kindergarten teachers’ organizational trust and job satisfaction, former studies do provide a theoretical basis for the correlation between organizational trust and intention to resign in rural kindergarten teachers. It was also found that self-efficacy can positively predict teachers’ job satisfaction [47]. The internal mechanism may be that teachers with a good sense of efficacy can better cope with challenging situations and are more committed to their own teaching work, thus, improving the retention rate of teachers [48]. Teachers’ self-efficacy also affects other social psychological factors influencing their motivation and performance, such as career ambition, career pressure, social recognition and respect for a career, ultimately leading to job satisfaction [30].

Although the four variables: job satisfaction, turnover intention, organizational trust, and self-efficacy, have appeared in pairs in existing studies, no one has developed a theoretical model to explain their interaction. This study may help to reveal the relationships among organizational trust, teaching efficacy, job satisfaction, and turnover intention among rural kindergarten teachers and investigate the specific mechanism of teaching efficacy and job satisfaction as mediating variables. Based on this analysis, the following hypotheses are made: (i) organizational trust in rural kindergarten teachers is negatively related to their turnover intention; (ii) the teaching efficacy in rural kindergarten teachers is negatively related to job satisfaction and turnover intention; (iii) organizational trust, teaching effectiveness and job satisfaction in rural kindergarten teachers are significantly positively correlated; (iv) teaching efficacy in rural kindergarten teachers plays a mediating role in the relationship between organizational trust and turnover intention; (v) job satisfaction mediates the relationship between organizational trust and turnover intention; (vi) organizational trust has an indirect effect on turnover intention via teaching efficacy and job satisfaction.

## 2. Materials and Methods

### 2.1. Participants and Procedure

By adopting the form of easy sampling, the study selected preschool teachers (excluding caregivers) aged 18 to 58 (M = 30.8, SD = 7.139) in 28 rural kindergartens in Shaanxi Province and Xinjiang Uygur Autonomous Region as the main research object. The average teaching experience was 6 years (M = 6.173, SD = 6.273), and the proportion of female teachers was 95.5%. Participants’ educational background levels were as follows: junior high school (*n* = 5, 1.6%), high school (*n* = 26, 8.3%), college degree (*n* = 129, 41.2%), bachelor’s degree (153, *n* = 48.9%) and master’s degree and above (0). With the consent of the kindergarten, the employee organizational trust scale, teacher self-efficacy scale (TSE) (simplified version), turnover intention scale, and teacher job satisfaction scale were distributed on-site. The total number of questionnaires distributed and returned was 330. All questionnaires were completed by preschool teachers themselves. Excluding incomplete and repeated questionnaires, 313 were valid, with an effective recovery rate of 94.8%. This study obtained ethics clearance from the Professors Committee of the Faculty of Education, Shaanxi Normal University. Before the investigation, teachers received informed consent, and the ethics committee approved all the processes of this study.

### 2.2. Methodology

#### 2.2.1. Organizational Trust Scale

The organizational trust scale was developed by Gabarro and Athos in 1976 [47]. This study used the trust scale in employers that was developed by Robinson in 1996 to select seven items from the original scale [49]. Chinese scholars revised it in 2007 and modified the original seven items into five items, e.g.,: “I believe my company is of great integrity”, “My company is always honest and trustworthy”, etc. The results showed that the MSS (measure of sampling suitability) of each item was greater than 0.85, which shows good reliability and validity [50]. A 5-point Likert-type scale was used to rate teachers, with options ranging from 1 point for “very inconsistent” to 5 points for “very consistent”. In this study, rural kindergarten teachers were taken as the research object, and the terms related to workplaces in the scale were changed while retaining the main words of the scale, such as: “I believe my kindergarten is very honest”, “my kindergarten is honest and credible” and so on. In this study, the coefficient of Cronbach’s α of the organizational trust scale was 0.939, which shows good internal consistency.

#### 2.2.2. Teacher Efficacy Scale

The teacher efficacy scale was developed by Tschannen–Moran in 2001 [51]. The brief version included 12 items. Then, Chinese scholars, Wu and Chim created a Chinese version [52]. The teachers choose their teacher efficacy level for each item on a Likert-type scale ranging from 1 (strongly disagree) to 5 (strongly agree). In this study, the Cronbach alpha coefficients of this scale were 0.823, which indicates good internal consistency. Example items included “I can build an effective classroom management model”, “I can convince young children that they can do well in kindergarten” etc.

#### 2.2.3. Job Satisfaction Scale

In 1951, Brayfield and Rothe developed the job satisfaction scale, which was widely used by many researchers [53]. However, the assessment of teacher job satisfaction also involves cognitive and judgmental processes, rather than simply measuring emotions. Based on this scale, Ho and Au developed a simple and convenient teacher job satisfaction scale (TTS) [54] (e.g., overall, being a kindergarten teacher fits my ideal). In this study, we created a Chinese version, which showed good reliability and validity (with the Cronbach alpha coefficients of this scale were 0.852). A Likert-type scale of 1 to 5 was used to rate participants (1 = very inconsistent to 5 = very consistent).

#### 2.2.4. Turnover Intentions Scale

The turnover intention scale originated from the research that Cropanzano and others have used to measure the possibility of individual resignation [55]. Later, through adaption by Konovsky and others, the turnover intention scale with three items was created [56]. Example items included “I might try changing my workplace next year”, “I often want to quit my job”, “I would like to change to another job if possible”. In a Likert-type scale, participants rated their consistency on a scale of 1 to 5 (1 = very inconsistent to 5 = very consistent). The higher total score meant a higher intention to leave. It showed good internal consistency in this study (with a Cronbach alpha coefficient of 0.82).

### 2.3. Data Analyses

We performed a descriptive data analysis (means and standard deviations), and then we conducted a Spearman correlation analysis to determine the relationships between variables. For the analysis of mediating effects, the bootstrap method was used with 5000 samples via the SPSS macro PROCESS [57]. This process generated a 95% confidence interval to evaluate the significance of the mediating role of teaching efficiency and job satisfaction on turnover intention. If there is no zero between the lower and upper confidence intervals (CIs), the indirect effects are significant.

## 3. Results

Since the four assessment scales this study used all came from the rural kindergarten teachers’ self-reports, there may be a common method bias. Therefore, we used confirmatory factor analysis to test whether common method bias exists. The results indicated there was no test common method bias effect (χ2/df = 10.758, CFI = 0.614, AGFI = 0.463, NFI = 0.593, RMSEA = 0.177).

Spearman correlations among variables were presented in Table 1, showing that the organizational trust in rural kindergarten teachers had a significant positive correlation with teaching efficacy, job satisfaction, and an apparent negative correlation with turnover intention. The teaching efficacy in rural kindergarten teachers was positively related to job satisfaction and negatively related to turnover intention. There was a negative correlation between job satisfaction and intention to leave for rural kindergarten teachers. Also, rural kindergarten teachers’ age and years of teaching experience negatively affected their intention to leave. Moreover, the results showed that there was no multicollinearity because no correlation coefficient exceeded 0.7.

We applied hierarchical linear analysis (HLA) to test the hypotheses proposed in the study. Four models were created, and the results are shown in Table 2. The results of model 1 indicate that organizational trust is negatively related to turnover intention. The negative correction between organizational trust and teaching efficacy is reported in model 2. Model 3 reveals that both teaching efficacy and teaching satisfaction are connected negatively with turnover intention. As shown in model 4, all independent variables have a negative connection with turnover intention.

**Figure 1:**
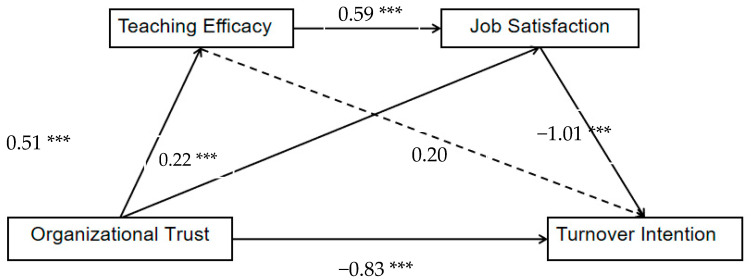
Mediator model examining the indirect relationship between organizational trust and turnover intention through teaching efficacy and job satisfaction. Note: Observations = 313. *** *p* < 0.001.

With 5000 bootstrapping resamples, the 95% CI of indirect effects was estimated using the bootstrapping method with bias-corrected confidence estimates [51]. The results are shown in Table 3 and Figure 1. The indirect effect of organizational trust on intention to leave is significant when teaching efficacy and job satisfaction are mediating variables. Even after accounting for mediator variables of two mediating roles, organizational trust still had a significant direct effect on intention to leave. The confidence intervals associated with the indirect effect of organizational trust and turnover intention did not contain zero (b = −0.23,95% CI = −0.40, −0.07,19.17%). The confidence intervals also did not contain zero when both teaching efficacy and job satisfaction played the role of mediator (b = 0.31, 95% CI = −0.47, −0.18, 24.60%).

## 4. Discussion

### 4.1. Analysis of the Relationship between Organizational Trust and Turnover Intention

This study found that the organizational trust in rural kindergarten teachers can negatively affect their intention to resign in a direct way. This is consistent with existing research results [58] confirming that employees who trust their organizations enjoy their work more and tend to pursue a longer-term career in the organization. According to organizational support theory and psychological safety theory, the goodwill and support of a group advances employees’ awareness of psychological safety [59]. A previous study suggested that employees have the courage to express their opinions and believe that their actions will be supported by the group when they are in a secure psychological atmosphere. Thus, they actively create value for their organization [60]. Furthermore, studies have shown that the impact of trust is much greater than that of emotional labor on turnover intention. That is to say, employees’ trust in the organization can offset the negative impact of emotional labor [61]. Therefore, in rural kindergartens, teachers who have higher organizational trust are less likely to experience turnover.

### 4.2. The Mediating Role of Job Satisfaction between Organizational Trust and Turnover Intention

The research shows that rural kindergarten teachers’ organizational trust had a positive effect on job satisfaction, and job satisfaction notably mediated the relationship between organizational trust and intention to resign, consistent with previous studies that reported the relationship between these variables in other environments [51]. On one hand, the social exchange theory suggests that employees with high organizational trust are more likely to be loyal to the organization and reward it with positive work behaviors [62]. Therefore, organizational trust can increase employees’ positive work attitude and behavior. With the enhancement of positive results, employees’ job satisfaction will naturally improve. As shown in previous studies, organizational characteristics are important factors affecting teachers’ job satisfaction, and teachers are more likely to have higher job satisfaction when working in a more supportive and autonomous organizational environment [63]. Teachers with high job satisfaction maintain an optimistic work attitude, actively improve work performance and take the initiative to solve problems. On the other hand, job satisfaction can negatively predict teachers’ turnover. When teachers are dissatisfied with their work, they tend to leave the organization, which has been confirmed in multiple studies [64,65]. Studies that tested the turnover model show that job satisfaction is a significant antecedent of turnover. If teachers work in a supportive psychological environment, they will experience beneficial outcomes, such as greater job satisfaction, which can stimulate their commitment to their organization [66]. They may then receive good supervisor feedback, obtain help from collaborators, and thus, create a more meaningful and enjoyable work environment, decreasing turnover [67].

In addition, when teachers’ job satisfaction acts as a mediating role, the work environment can also impact teachers’ professional commitment, professional autonomy and burnout, which have an significant effect on resignations [68]. For this reason, job satisfaction is an essential mediator between organizational trust and turnover. Because of the shortage of teachers in rural Chinese kindergartens, there are few conflicts and mutual doubts among teachers. As a result, rural kindergarten teachers typically have simpler and more harmonious interpersonal relationship, which results in low conflict levels and high job satisfaction. Because of this, they express more positive emotions toward the organization and maintain a positive attitude towards work, with a corresponding lower turnover rate.

### 4.3. The Mediating Role of Teaching Efficacy between Organizational Trust and Turnover Intention

Moreover, this study shows that organizational trust predicted the teaching efficacy in rural kindergarten teachers, confirming previous research. An organizational culture of trust and support is fundamental to a healthy and productive work environment [69], which is conducive to employees’ active involvement, ability realization and performance improvement. Therefore, a strong sense of organizational trust improves teaching efficacy. However, the mediating role of teaching efficacy between organizational trust and turnover intention is not significant, although teachers with higher teaching efficacy have low resignation rates. This is inconsistent with other research that reports on factors that have an impact on turnover via efficacy [70,71]. A possible reason is that rural preschool teachers have a smaller range of job choices than urban teachers. Even if they have higher abilities, achieving upward mobility in a specific area is challenging. Therefore, teaching efficacy does not have a significant influence on rural preschool teachers’ decisions to resign.

### 4.4. The Mediating Chain Effect of Teaching Efficacy and Job Satisfaction

The study found a notable chain mediating model: rural kindergarten teachers’ teaching efficacy and job satisfaction mediate between organizational trust and turnover intention. This is similar to Kim and Kao’s suggestion that burnout and emotional exhaustion affect turnover intention in the American child welfare industry [72].

Furthermore, teachers with higher teaching efficacy have higher job satisfaction. Teaching efficacy represents the perceived distance between individual ability and job requirements, which is a subjective index for measuring competence. It not only affects educational behavior and educational outcomes but also job motivation and work environment [73]. Job satisfaction is an individuals’ psychological preference to work, which can directly affect working engagement [74] and motivation to leave [75]. Teachers have stronger job satisfaction when they have higher expectations of their own achievement. Therefore, the higher the teaching efficacy, the higher the job satisfaction.

## 5. Conclusions

This study investigated the relationship of organizational trust, teaching efficacy, job satisfaction, and turnover intention in rural kindergarten teachers. We found that: (i) the organizational trust level in rural kindergarten teachers can negatively predict their turnover intention, (ii) teaching efficacy and job satisfaction have a negative correlation with teachers’ turnover intention, and (iii) organizational trust is positively related to teaching efficacy and job satisfaction, and (iv) teaching efficacy and job satisfaction played positive chain mediating roles between organizational trust and turnover intention.

More important, the outbreak of COVID-19 has had a huge impact on the normal life and work of individuals [76]. Similar to other professions [77], kindergarten teachers have experienced a career crisis during the COVID-19 pandemic. In this study, these results provided a valuable reference for understanding and solving the problem of rural kindergarten teachers’ turnover. In addition, the problems of uneven development and educational inequality are widespread in developing countries. Given the poor working environment, heavy workload and low pay for rural kindergarten teachers, how to effectively improve the stability of the rural kindergarten teachers is a common concern in other developing countries.

The present study may also provide inspiration for Chinese rural kindergartens to build a climate of trust and optimize teacher management. Consequently, kindergartens should support teachers’ work and provide them with a platform for communication, cooperation and learning to create a democratic work atmosphere, so that teachers’ efficacy and job satisfaction are improved, and teachers’ tendency to resign will be reduced. As a result, these findings are essential for kindergarten teachers in the post-COVID era who need organizational support and guidance.

## 6. Limitations

The study still has the following limitations. First, it employed convenience sampling to collect data from two provinces in China, which may limit the generalizability of findings. Second, the study adopted quantitative research methods, and the data collection is primarily through self-report, resulting in some inconsistencies in the results. Third, the methods of this study were cross-sectional. Future research could include a longitudinal study to eliminate reverse causality and enhance the reliability of results. Finally, we identify the teacher efficacy and job satisfaction in rural kindergarten teachers as mediating variables on the impact of organizational trust and turnover intention. If there are other more crucial mediating variables, more research can be conducted in the future.

## Figures and Tables

**Table 1:** The inter-correlations of all the studied variables.

Spearman Rho	Means (SD)	1	2	3	4	5	6	7	8	9
1. Age	30.80 7.14	1								
2. Teaching age	6.17 6.27	0.60 **	1							
3. Gender	_	−0.04	0.01	1						
4. Class	_	0.07	0.12	−0.07	1					
5. Degree	3.78 0.50	−0.03	0.11	−0.07	0.06	1				
6. Organizational Trust	4.45 0.53	0.08	0.08	0.06	0.04	−0.07	1			
7. Teaching Efficacy	4.21 0.48	−0.14 *	−0.16 **	−0.05	0.06	0.04	−0.36 **	1		
8. Job Satisfaction	3.40 0.60	0.01	0.10	0	0.05	0.03	0.57 **	−0.27 **	1	
9. Turnover Intention	1.14 0.52	0.05	0.04	0.05	−0.03	−0.04	0.44 **	−0.44 **	0.55 **	1

Note: Observations = 313. * *p* < 0.05.** *p* < 0.01.

**Table 2:** The results of regression analysis (models 1–3).

Regression		Overall Fit Index			Regression Coefficient Significance	
Dependent Variable	Independent Variable	R	R^2^	F	β	t
1 Turnover Intention		0.39	0.15	19.48 ***		
	Age				−0.08	−2.13 ***
	Working Age				−0.03	−0.84
	Organization Trust				−1.26	−6.56 ***
2 Teaching Efficacy		0.56	0.31	47.04 ***		
	Age				−0.01	−1.23
	Working Age				0.01	1.60
	Organization Trust				0.51	11.58 ***
3 Teaching satisfaction		0.57	0.32	37.50 ***		
	Age				0.02	1.90 ***
	Working Age				−0.01	−0.95
	Organization Trust				0.22	3.19 ***
	Teaching Efficacy				0.59	7.92 ***
4 Turnover Intention		0.50	0.23	19.99 ***		
	Age				−0.07	−1.74
	Working Age				−0.03	−1.03
	Organization Trust				−0.83	−3.73 ***
	Teaching Efficacy				0.20	0.78
	Teaching satisfaction				−1.01	−5.68 ***

Note: Observations = 313. *** *p* < 0.001.

**Table 3:** The results of mediation analysis.

	b	Boot Standard Error	Boot CI		Relative Mediation Effect
			Lower limit	Upper Limit	
Total Indirect Effect	−0.43	0.14	−0.72	−0.15	34.13%
Indirect Effect I	0.10	0.12	−0.13	0.30	
Indirect Effect II	−0.23	0.08	−0.40	−0.07	19.17%
Indirect Effect III	−0.31	0.07	−0.47	−0.18	24.60%

Notes: Indirect Effect I = organizational trust- > teaching efficacy- > turnover intention; indirect effect II = organizational trust- > job satisfaction- > turnover intention; indirect effect III = organizational trust- > teaching efficacy- > job satisfaction- > turnover intention.

## Data Availability

Access to individual raw data is protected as private information and subject to a five-year embargo policy. The data will be available from the author upon reasonable request after 2027.
